# Causal relationships between type 2 diabetes, glycemic traits and keratoconus

**DOI:** 10.3389/fmed.2023.1264061

**Published:** 2023-11-06

**Authors:** Xueying Zhu, Dan Cheng, Kaiming Ruan, Meixiao Shen, Yufeng Ye

**Affiliations:** National Clinical Research Center for Ocular Diseases, Eye Hospital, Wenzhou Medical University, Wenzhou, China

**Keywords:** Mendelian randomization, keratoconus, fasting glucose, proinsulin levels, type 2 diabetes, genome-wide association study

## Abstract

**Purpose:**

The relationship between diabetes mellitus and keratoconus remains controversial. This study aimed to assess the potential causal relationships among type 2 diabetes, glycemic traits, and the risk of keratoconus.

**Methods:**

We used a two-sample Mendelian randomization (MR) design based on genome-wide association summary statistics. Fasting glucose, proinsulin levels, adiponectin, hemoglobin A1c (HbA1c) and type 2 diabetes with and without body mass index (BMI) adjustment were used as exposures and keratoconus was used as the outcome. MR analysis was performed using the inverse-variance weighted method, MR-Egger regression method, weighted-mode method, weighted median method and the MR-pleiotropy residual sum and outlier test (PRESSO).

**Results:**

Results showed that genetically predicted lower fasting glucose were significantly associated with a higher risk of keratoconus [IVW: odds ratio (OR) = 0.382; 95% confidence interval (CI) = 0.261–0.560; *p* = 8.162 × 10^−7^]. Genetically predicted lower proinsulin levels were potentially linked to a higher risk of keratoconus (IVW: OR = 0.739; 95% CI = 0.568–0.963; *p* = 0.025). In addition, genetically predicted type 2 diabetes negatively correlated with keratoconus (IVW: BMI-unadjusted: OR = 0.869; 95% CI = 0.775–0.974, *p* = 0.016; BMI-adjusted: OR = 0.880, 95% CI = 0.789–0.982, *p* = 0.022). These associations were further corroborated by the evidence from all sensitivity analyses.

**Conclusion:**

These findings provide genetic evidence that higher fasting glucose levels are associated with a lower risk of keratoconus. However, further studies are required to confirmed this hypothesis and to understand the mechanisms underlying this putative causative relationship.

## 1. Introduction

Keratoconus is the most common type of ectatic corneal disease. It is defined as progressive corneal thinning causing corneal protrusion, uneven astigmatism, and impaired vision, which can result in legal blindness if left untreated (1–4). The incidence of keratoconus varies from 1:2000 cases as documented in 1986 (5) to 1:375 cases recorded in 2016 (6). The increasing prevalence of keratoconus underscores the significance of identifying the risk factors associated with its development.

Keratoconus is a complex disease that involves a mixture of environmental and genetic factors (7, 8), but its exact etiology remains elusive. Studies on the effects of diabetes mellitus (DM) on the incidence of keratoconus differ significantly. Seiler et al. (9) was the first to demonstrated that DM is a protective factor against keratoconus. Three other studies also found similar results, with a considerably lower rate of DM in patients with keratoconus than in non-keratoconus controls (10–12). However, some conflicting studies have shown that the prevalence of DM is greater in patients with keratoconus than in the control group (13, 14). A recent meta-analysis by Hashemi et al. (15) revealed an unclear correlation between DM and keratoconus. The reasons for these contradictory study results may be due to innate biases or confounders in the observational studies, such as clinic-based case-control recruitment, small sample sizes, reverse causality, and demographic trait heterogeneity.

Mendelian randomization (MR) is a reliable approach for estimating the causal contribution of reported genetic instrumental variables to the disease outcomes of interests (16, 17). MR is less vulnerable to the impact of reverse causality or confounding factors than conventional observational research (17, 18). We used this MR study to determine the causal effect of genetically predicted type 2 diabetes and multiple glycemic traits on the risk of keratoconus in European populations.

## 2. Methods

### 2.1. Study design

**Figure 1:**
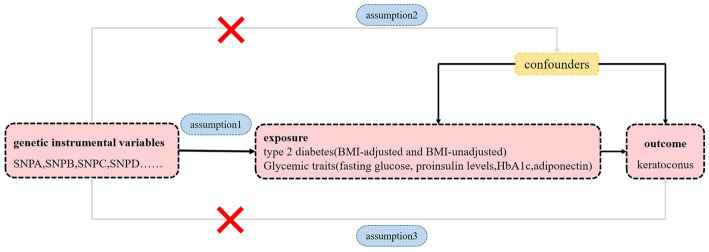
Diagram of MR principles investigating the causal relationship between type 2 diabetes, glycemic traits and keratoconus. Instrumental variable assumptions: Assumption 1: genetic instrumental variables must be closely linked to exposure. Assumption 2: genetic instrumental variables are irrelevant to any confounders influencing the exposure-outcome link. Assumption 3: genetic instrumental variables affect outcomes merely through their effect on the exposure.

To estimate the causal relationships between exposure to type 2 diabetes and glycemic traits and the risk of keratoconus, we performed a two-sample MR analysis based on summary statistical data from genome-wide association study (GWAS). We used type 2 diabetes with and without adjustment for body mass index (BMI) and four glycemic traits: fasting glucose, proinsulin levels, adiponectin, and hemoglobin A1c (HbA1c) as exposures. Keratoconus was used as an outcome measure. Applying MR analyses requires the following three essential assumptions (Figure 1): (1) genetic instrumental variables must be closely linked to exposure; (2) genetic instrumental variables are irrelevant to any confounders influencing the exposure-outcome link and (3) genetic instrumental variables affect outcomes merely through their effect on exposure.

This study adhered to the principles of the Declaration of Helsinki. This study was approved by the Institutional Review Board of the Eye Hospital of Wenzhou Medical University as it used only publicly available data.

### 2.2. Genetic instruments for type 2 diabetes and glycemic traits

Intake of diets high in sugar are associated with insulin resistance, hyperglycemia and obesity (19, 20). In the present, insulin resistance (adiponectin levels), hyperglycemia (HbA1c and fasting glucose), and β-cell dysfunction (proinsulin levels) were selected as glycemic traits. GWAS summary datasets of adiponectin, fasting glucose and proinsulin levels were acquired from the MRC-IEU OpenGWAS project software^1^ (21). These three glycemic traits were derived from different GWASs, including adiponectin (GWASID: ieu-a-1; *n* = 39,883) (22), fasting glucose (GWASID: ieu-b-114; *n* = 133,010) (23) and proinsulin levels (GWASID: ebi-a-GCST001212; *n* = 10,701) (19). The selections of genetic instrumental variables for HbA1c were based on a large GWAS meta-analysis involving 123,665 participants of European ancestry without diabetes (24).^2^ The summary statistics for type 2 diabetes were obtained from a meta-analysis of GWAS of European ancestry (25). The studies conducted meta-analyses with and without adjustment for BMI. Single nucleotide polymorphisms (SNPs) for type 2 diabetes were obtained from the MRC-IEU OpenGWAS project (21), including type 2 diabetes adjusted for BMI (GWASID: ebi-a-GCST007516; *n* = 298,957) and unadjusted for BMI (GWASID: ebi-a-GCST007517; *n* = 298,957) (25). Detailed information of type 2 diabetes and every glycemic trait is presented in Table 1. All SNPs chosen as instrumental variants were strongly associated with the relevant exposure and reached Genome-wide significance (*p* < 5 × 10^−8^). The variants were then trimmed using linkage disequilibrium (*r* ≤ 0.001 within a distance of 10,000 kb for variants at the same locus). The *F*-statistic was used to quantify the instrumental strength for each candidate SNP and SNPs with *F* > 10 were retained. Effect estimates of SNP associated with type 2 diabetes were categorized as unadjusted and adjusted for BMI. The final independent SNPs determined as genetic instruments for each exposure were shown in Supplementary Tables S1, S2.

**Table 1:** Description of GWAS summary statistics for type 2 diabetes and glycemic traits.

Phenotype	PMID	Accession	Sample size	Number of SNPs	Population ethnicity	Study
Adiponectin	22479202	The MRC IEU (GWASID: ieu-a-1)^a^	39,883	2,675,209	Mixed	Dastani et al. (22)
Fasting glucose	22885924	The MRC IEU (GWASID: ieu-b-114)^a^	133,010	64,432	European	Scott et al. (23)
Proinsulin levels	21873549	The MRC IEU (GWASID: ebi-a-GCST001212)^a^	10,701	2,479,861	European	Strawbridge et al. (19)
HbA1c	28898252	The MAGIC website^b^	123,665	2,586,698	European	Wheeler et al. (24)
Type 2 diabetes (adjusted for BMI)	29632382	The MRC IEU (GWASID: ebi-a-GCST007516)^a^	298,957	131,045	European	Mahajan et al. (25)
Type 2 diabetes (unadjusted for BMI)	29632382	The MRC IEU (GWASID: ebi-a-GCST007517)^a^	298,957	190,208	European	Mahajan et al. (25)

a The MRC IEU OpenGWAS database (https://gwas.mrcieu.ac.uk/).

b The MAGIC website (www.magicinvestigators.org).

### 2.3. GWAS summary statistics for keratoconus

Genetic variants associated with keratoconus were acquired from the first large scale GWAS. Importantly, we used the first stage meta-analysis comprising of 2,116 cases and 24,626 controls of European ancestry (26).^3^

### 2.4. Statistical analysis

For each relevant exposure, we performed two-sample MR analyses using R version 4.1.0 (R Foundation for Statistical Computing, Vienna, Austria). The methods based on the TwoSampleMR version 0.5.6 R package included the inverse-variance weighted (IVW) (27), weighted mode (28), weighted median (29), MR pleiotropy residual sum and outlier (MR-PRESSO) test (30), and MR-Egger regression methods (31). IVW is a major method for assessing the relationships among type 2 diabetes, glycemic traits, and keratoconus (32). Causal analysis of type 2 diabetes and keratoconus were categorized as unadjusted and adjusted for BMI. If the IVW approach indicated an association (*p* < 8.333 × 10^−3^ = 0.05/6, taking into account multiple testing for six exposures and one outcome) and the five MR methods had effects in a consistent direction, the results were regarded as statistically significant. *p* < 0.05, but greater than the significance threshold after Bonferroni correction; the five MR methods had effects in a consistent direction suggesting a potential association. For sensitivity analysis, the Egger intercept calculation (31), MR-PRESSO global test (30), the Cochran’s *Q* test (33), and the leave-one-out analysis (28) were used to estimate the strength of these identified associations.

## 3. Results

### 3.1. MR analysis of fasting glucose and risk of keratoconus

**Figure 2:**
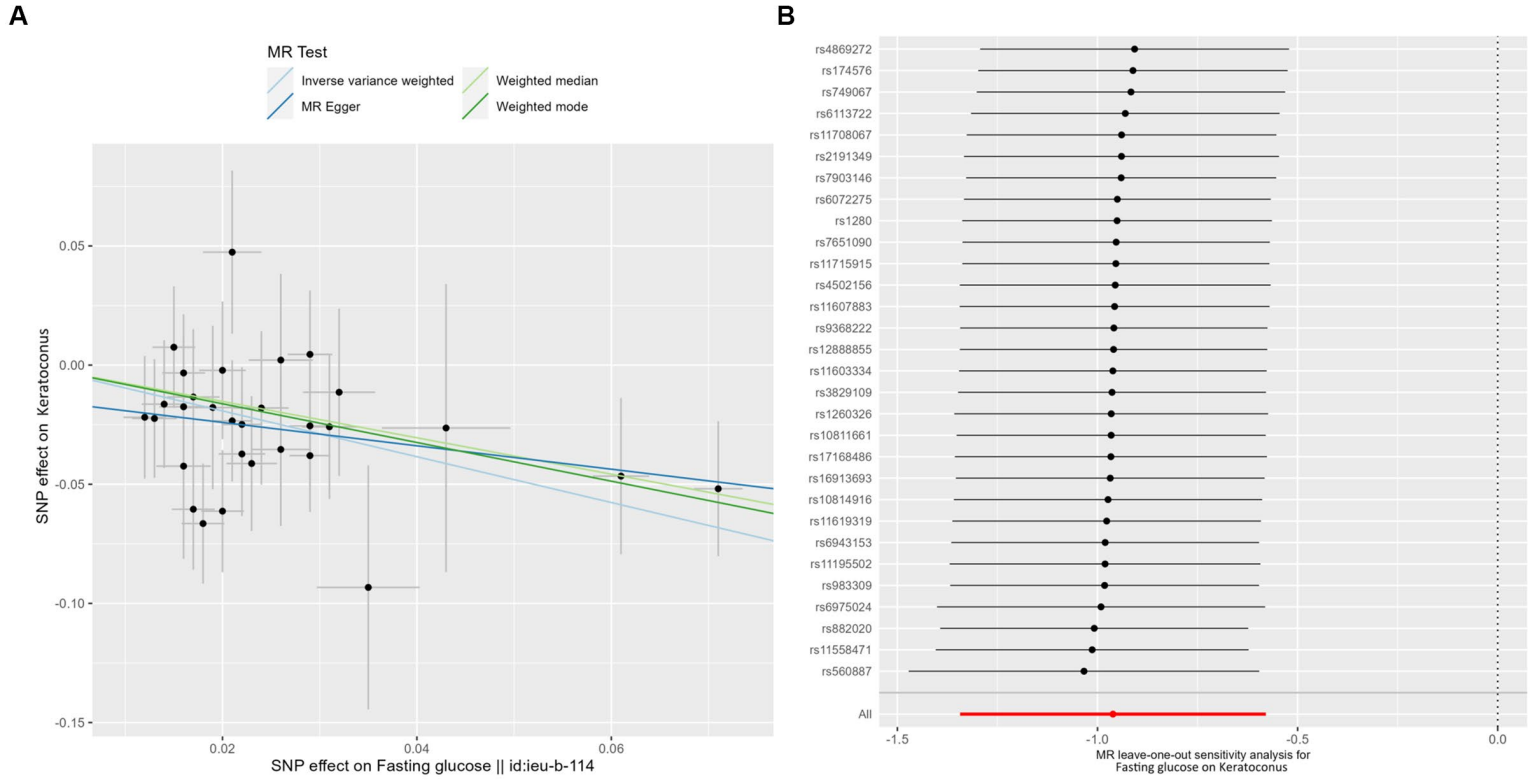
MR analysis and leave-one-out analysis of the causal effect of fasting glucose on keratoconus. **(A)** Scatter plots for MR analyses of the causal effect of fasting glucose on keratoconus. Each line shows the slope corresponding to the estimated MR effect each method. **(B)** Leave-one-out analysis of the causal effect of fasting glucose on keratoconus. Every black dot represents the IVW MR method applied to estimate the causal effect of fasting glucose on keratoconus, with particular variant excluded from the analysis. The red point represents the IVW estimate using all SNPs.

The results showed that a genetically predicted higher fasting glucose level was considerably associated with a lower risk of keratoconus (Table 2): IVW [odds ratio (OR) = 0.382; 95% confidence interval (CI) = 0.261–0.560; *p* = 8.162 × 10^−7^]. The uniform direction of the fasting glucose level effect indicates that it has a protective impact against keratoconus. The scatter plots of the MR analysis in Figure 2A demonstrate the effective levels of associations between fasting glucose and keratoconus. Even after Bonferroni adjustment, the IVW and MR PRESSO results remained statistically significant (*p <* 8.333 × 10^−3^). The Egger intercept indicated no horizontal pleiotropy effects (*p* = 0.220 > 0.05). Heterogeneity from the Cochran’s *Q* test was not statistically significant (*p* = 0.858 > 0.05). No outliers were detected in the leave-one-out analysis (Figure 2B). Furthermore, no horizontal pleiotropic outliers were identified to distort these results using MR-PRESSO (global test *p* = 0.862 > 0.05). Collectively, these results support the inverse causal relationship between low fasting glucose levels and keratoconus occurrence.

**Table 2:** Mendelian randomization estimates for associations between type 2 diabetes, glycemic traits and keratoconus.

Method	No. of SNPs	Forest plots	OR	95% CI	*p*	*p*-het	*p*-intercept	*p*-global
**Adiponectin**								
IVW	13	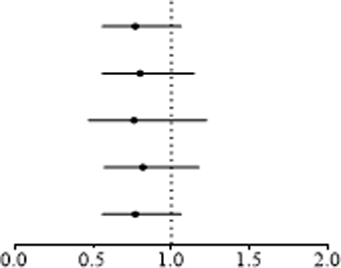	0.771	0.560–1.063	0.113	0.008		
Weighted median	13		0.800	0.559–1.145	0.223			
MR-Egger	13		0.762	0.473–1.226	0.286		0.942	
Weighted mode	13		0.820	0.573–1.175	0.266			
MR-PRESSO	13		0.771	0.560–1.063	0.139			0.036
**Fasting glucose**								
IVW	30	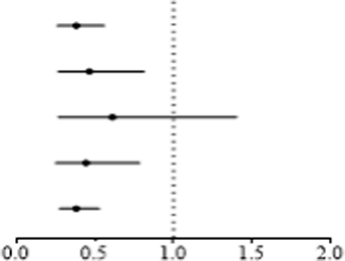	0.382	0.261–0.560	8.162 × 10–7	0.858		
Weighted median	30		0.466	0.267–0.815	6.298 × 10–3			
MR-Egger	30		0.612	0.267–1.402	0.256		0.220	
Weighted mode	30		0.444	0.251–0.786	0.011			
MR-PRESSO	30		0.382	0.276–0.529	2.836 × 10–6			0.862
**HbA1c**								
IVW	37	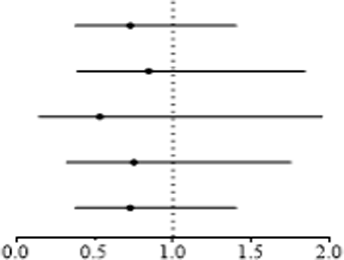	0.728	0.378–1.404	0.344	0.004		
Weighted median	37		0.846	0.389–1.843	0.692			
MR-Egger	37		0.534	0.146–1.953	0.349		0.588	
Weighted mode	37		0.751	0.321–1.753	0.463			
MR-PRESSO	37		0.728	0.378–1.404	0.350			0.005
**Proinsulin levels**								
IVW	7	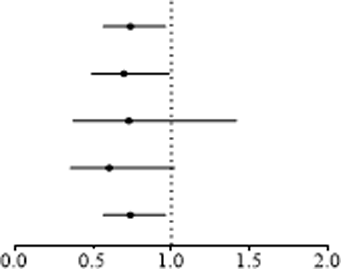	0.739	0.568–0.963	0.025	0.337		
Weighted median	7		0.697	0.492–0.986	0.043			
MR-Egger	7		0.729	0.375–1.416	0.393		0.963	
Weighted mode	7		0.604	0.357–1.021	0.129			
MR-PRESSO	7		0.739	0.568–0.963	0.066			0.335
**Type 2 diabetes (adjusted for BMI)**								
IVW	54	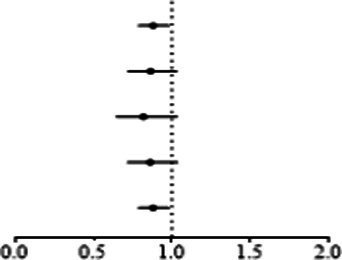	0.880	0.789–0.982	0.022	0.339		
Weighted median	54		0.864	0.723–1.031	0.105			
MR-Egger	54		0.819	0.648–1.035	0.100		0.497	
Weighted mode	54		0.863	0.719–1.036	0.104			
MR-PRESSO	54		0.880	0.789–0.982	0.026			0.370
**Type 2 diabetes (unadjusted for BMI)**								
IVW	51	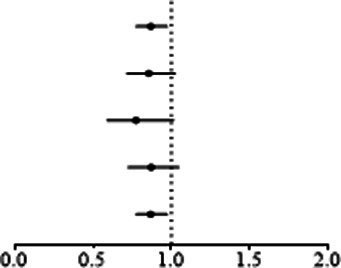	0.869	0.775–0.974	0.016	0.208		
Weighted median	51		0.858	0.717–1.026	0.091			
MR-Egger	51		0.774	0.591–1.014	0.069		0.359	
Weighted mode	51		0.872	0.726–1.047	0.121			
MR-PRESSO	51		0.869	0.775–0.974	0.020			0.225

SNP, single nucleotide polymorphism; OR, odds ratio; CI, confidence interval; MR, Mendelian randomization; IVW, inverse-variance weighted; PRESSO, pleiotropy residual sum and outlier; *p*-het, *p*-value for heterogeneity using Cochran’s *Q* test; *p*-intercept, *p*-value for MR-Egger intercept; *p*-global, *p*-value for MR-PRESSO global test.

### 3.2. MR analysis of proinsulin levels and risk of keratoconus

**Figure 3:**
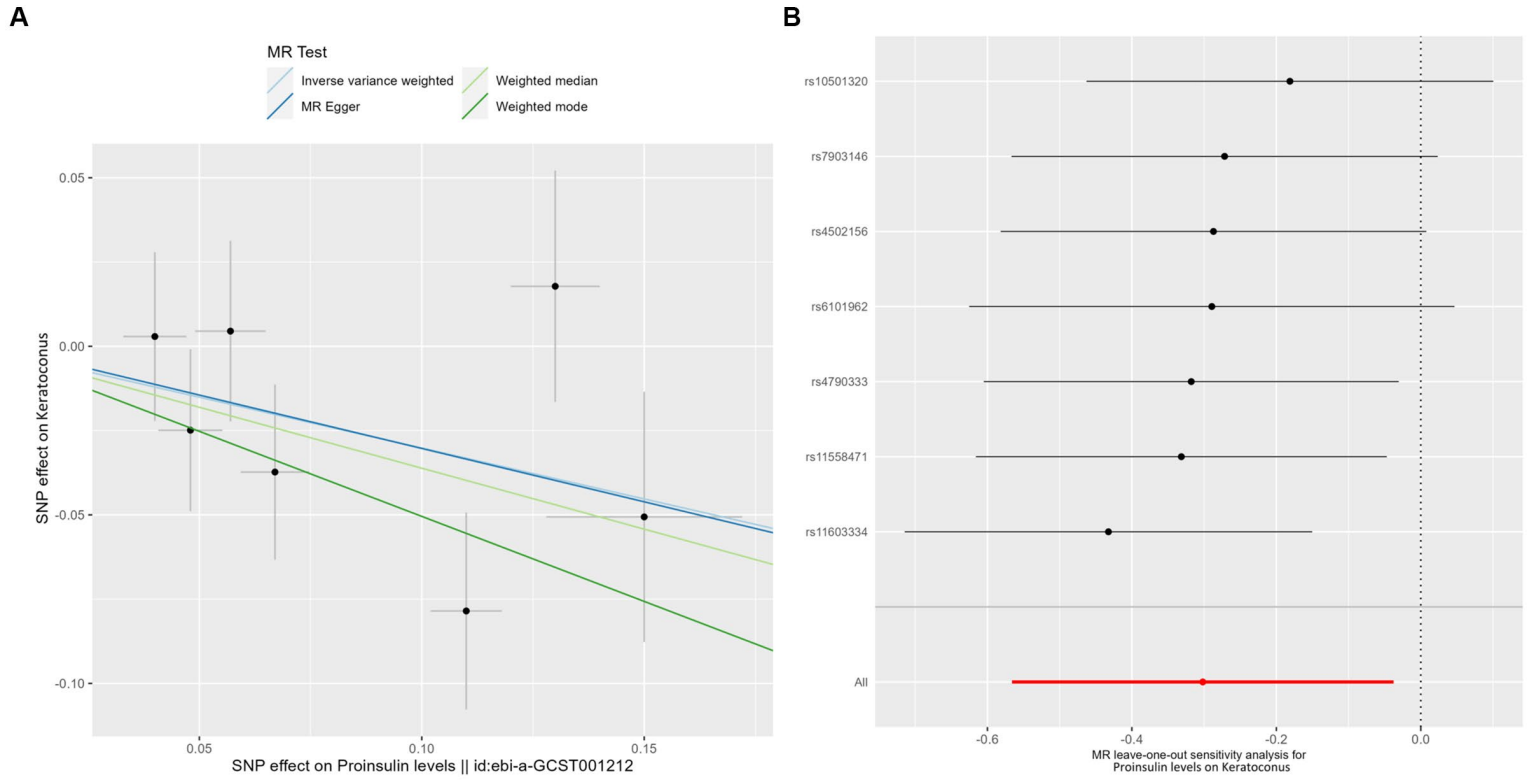
MR analysis and leave-one-out analysis of the causal effect of proinsulin levels on keratoconus. **(A)** Scatter plots for MR analyses of the causal effect of proinsulin levels on keratoconus. Each line shows the slope corresponding to the estimated MR effect each method. **(B)** Leave-one-out analysis of the causal effect of proinsulin levels on keratoconus. Every black dot represents the IVW MR method applied to estimate the causal effect of proinsulin levels on keratoconus, with particular variant excluded from the analysis. The red point represents the IVW estimate using all SNPs.

Genetically predicted proinsulin levels were found to be potentially inversely linked to the incidence of keratoconus (Table 2): IVW (OR = 0.739; 95% CI = 0.568–0.963; *p* = 0.025). The proinsulin levels effect in consistent direction indicated that it has a potential protective effect against keratoconus. The scatter plots of the MR analysis in Figure 3A demonstrate the effective level of the associations between proinsulin levels and keratoconus. The MR-Egger intercept provided no support for directional pleiotropy (*p* = 0.693 > 0.05). Heterogeneity from Cochran’s *Q* test was not statistically significant (*p* = 0.337 > 0.05). No outliers were detected in the leave-one-out sensitivity analysis (Figure 3B). Furthermore, no horizontal pleiotropic outliers were found in the MR-PRESSO test to distort these results (global test *p* = 0.335 > 0.05). In combination, these results support a potential negative relationship between low proinsulin levels and the occurrence of keratoconus.

### 3.3. MR analysis of type 2 diabetes and risk of keratoconus

**Figure 4:**
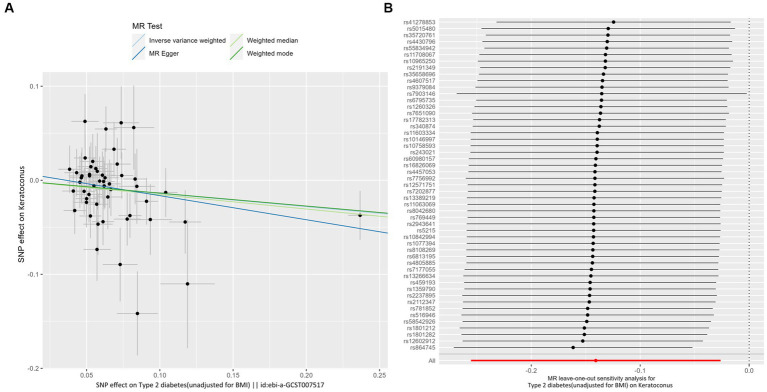
MR analysis and leave-one-out analysis of the causal effect of type 2 diabetes unadjusted for BMI on keratoconus. **(A)** Scatter plots for MR analyses of the causal effect of type 2 diabetes unadjusted for BMI on keratoconus. The slope of each line corresponds to the estimated MR effect per method. **(B)** Leave-one-out analysis of the causal effect of type 2 diabetes unadjusted for BMI on keratoconus. Each black point represents the IVW MR method applied to estimate the causal effect of type 2 diabetes unadjusted for BMI on keratoconus, excluding that particular variant from the analysis. The red point represents the IVW estimate using all SNPs.

**Figure 5:**
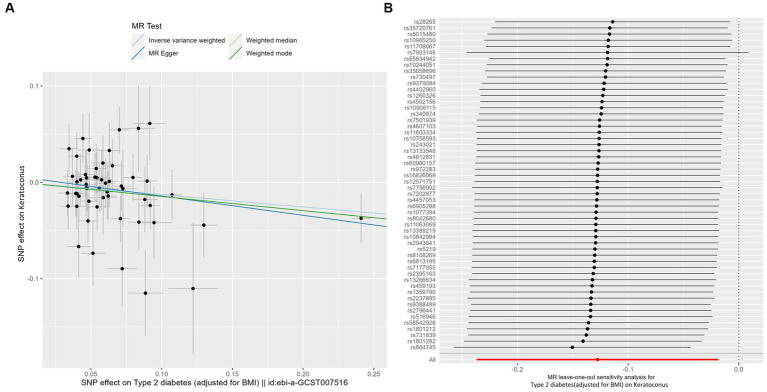
MR analysis and leave-one-out analysis of the causal effect of type 2 diabetes adjusted for BMI on keratoconus. **(A)** Scatterplots for MR analyses of the causal effect of type 2 diabetes adjusted for BMI on keratoconus. The slope of each line corresponds to the estimated MR effect per method. **(B)** Leave-one-out analysis of the causal effect of type 2 diabetes adjusted for BMI on keratoconus. Each black point represents the IVW MR method applied to estimate the causal effect of type 2 diabetes adjusted for BMI on keratoconus, excluding that particular variant from the analysis. The red point represents the IVW estimate using all SNPs.

IVW analyses showed that genetically predicted levels of type 2 diabetes were potentially inversely associated with keratoconus (Table 2): BMI-adjusted: OR = 0.880; 95% CI = 0.789–0.982; *p* = 0.035; BMI-unadjusted: OR = 0.869; 95% CI = 0.775–0.974; *p* = 0.016. The uniform direction of the impact of type 2 diabetes indicated that it has a potential protective effect against keratoconus. The scatter plots of the MR analyses in Figure 4A (BMI-unadjusted) and Figure 5A (BMI-adjusted) demonstrate the effective level of the associations between type 2 diabetes and keratoconus. The MR-Egger intercept test did not show any directional pleiotropy (BMI-unadjusted: *p* = 0.359; BMI-adjusted: *p* = 0.497). Heterogeneity from Cochran’s *Q* test was not statistically significant (BMI-unadjusted: *p* = 0.208; BMI-adjusted: *p* = 0.339). The leave-one-out analysis identified no outliers (Figure 4B: BMI-unadjusted; Figure 5B: BMI-adjusted). Furthermore, MR-PRESSO analysis did not identify horizontal pleiotropic variants, distorting these results with global test *p* > 0.05 (BMI-unadjusted: *p* = 0.225; BMI-adjusted: *p* = 0.370). All together, these results support type 2 diabetes is potentially inversely associated with the incidence of keratoconus.

## 4. Discussion

To the best of our knowledge, this study is the first to describe the causal associations between type 2 diabetes, glycemic traits, and the risk of keratoconus using MR analysis. This study provided evidence of a causal relationship between higher fasting glucose levels and a lower incidence of keratoconus. Meanwhile, our findings imply that increased proinsulin levels potentially decrease the risk of keratoconus. In addition, type 2 diabetes was related to a decreased incidence of keratoconus irrespective of whether type 2 diabetes was adjusted or unadjusted for BMI.

The relationship between DM and keratoconus remains controversial owing to the conflicting results from multiple studies. Several studies have shown an inverse relationship between DM and the risk of keratoconus, suggesting a protective role in the development of keratoconus (9–12). In contrast, other studies that have demonstrated a positive correlation or no association between DM and keratoconus (2, 13, 34, 35). In the present study, we discovered that higher fasting glucose levels were associated with a lower risk of keratoconus, but there was no discernible effect of HbA1c levels. HbA1c represents the average level of blood glucose during the previous two to 3 months (36). Previous studies have reported that genetically predicted fasting glucose and HbA1c levels are inconsistent and there is significant disagreement in the diagnosis of DM (37–39). Our findings indicate that adiponectin does not protect against keratoconus. Adiponectin, an endocrine hormone mainly generated and released by adipocytes, has no causal impact on glucose homeostasis and type 2 diabetes, and correlations among them in observational designs may be caused by the underlying confounding factors reported by Chen et al. (40). The precursor of insulin, proinsulin, is secreted in increased quantities when pancreatic β-cells are stressed, and prior studies have demonstrated that increased proinsulin could serve as a signal to those with prediabetes (41). In particular, our results indicate that proinsulin serves as a protective factor for keratoconus and increases proinsulin levels, thereby reducing the risk of keratoconus. An elevated proinsulin-to-insulin ratio in the blood has been postulated as a potential marker of type 2 diabetes for more than 20 years (42). Notably, our study supported an accordant trend that is elevated fasting glucose, increased proinsulin levels and type 2 diabetes are all negatively related to the incidence of keratoconus.

These interesting findings raise concerns regarding how and why fasting glucose levels affect keratoconus. These mechanisms may include modifications in corneal biomechanics and collagen crosslinking, changes in the extracellular matrix structure, oxidative stress, proteolytic activity, and increased inflammation (43). Here, we postulate on a theoretic level that DM protects keratoconus by altering the biomechanics of the cornea and increasing collagen cross-linking. A few studies have shown that the expression and activity of LOX, a copper amine oxidase that triggers the collagen cross-linking (44), increased in skin collagen (45, 46), rat retinal endothelial cells (46), and ARPE-19 cells (47) under hyperglycemic states. Moreover, the cornea has detected to contain the Lysyl oxidase (LOX) enzyme (48). Therefore, if LOX expression is upregulated in corneal cells under hyperglycemia, this could explain why individuals with DM have a lower risk of developing keratoconus (43). In addition, diabetes may protect against keratoconus through non-enzymatic approaches. Advanced glycation end product (AGE)-mediated crosslinking increases corneal stromal collagen cross-linking, thus strengthening corneal stiffness (44). Nevertheless, the underlying mechanisms of this association need to be assessed more comprehensively.

The use of MR analysis to evaluate large-scale databases using standard procedures is one of the main advantages of this study. The MR method is far less vulnerable to biases or confounders than observational studies. However, this study has some limitations. First, because individuals of European ancestry participated in this study, our findings applying to other races needs further investigation. Second, further laboratory research is required, because the effects of fasting glucose on keratoconus development have not yet been experimentally explored. Third, we only took into account BMI when selecting the instrumental variables for type 2 diabetes in this two-sample MR design. However, no discernible differences were observed between the unadjusted and adjusted BMI models, making multivariate MR a more practical method.

Our findings concluded that the relationship between low fasting levels and a high risk of keratoconus is causal. Furthermore, higher proinsulin levels decrease the risk of keratoconus. These findings, utilized in clinics, indicate that a rational sugar diet strategy may be favorable in controlling keratoconus. However, further studies are required to confirm this hypothesis and to comprehend the mechanisms underlying this putative causative relationship.

## Data availability statement

The original contributions presented in the study are included in the article/Supplementary material, further inquiries can be directed to the corresponding author.

## Author contributions

XZ: Writing – original draft, Writing – review & editing. DC: Writing – review & editing. KR: Writing – review & editing. MS: Writing – review & editing. YY: Writing – review & editing.

## Footnotes

^1^https://gwas.mrcieu.ac.uk/

^2^www.magicinvestigators.org/downloads

^3^https://www.ebi.ac.uk/gwas/
